# High-resolution manometry in the elderly: patterns of functional dysphagia, achalasia subtypes, and clinical predictors

**DOI:** 10.3389/fphys.2026.1961993

**Published:** 2026-10-14

**Authors:** Deepanjan Dey, Rahul Manral, Manish Manrai, Aditya Jayapalan, Pradeep Behal, Santosh Kumar Singh

**Affiliations:** 1 Department of Physiology, Armed Forces Medical College, Pune, India; 2 Department of Gastroenterology, Armed Forces Medical College, Pune, India; 3 Armed Forces Medical College, Pune, India; 4 Department of Geriatrics, Armed Forces Medical College, Pune, India; 5 Department of Internal Medicine, Armed Forces Medical College, Pune, India

**Keywords:** ageing, dysphagia (swallowing disorder), esophageal motility disorder, high-resolution manometry, lower esophageal sphincter

## Abstract

**Background:**

Dysphagia in the elderly is often attributed to ageing without objective functional testing. We describe high-resolution manometry (HRM) metrics and esophageal motility disorder (EMD) classification in a consecutive geriatric cohort to inform referral and management.

**Methods:**

In this cross-sectional study we enrolled 176 consecutive patients aged >65 years presenting with esophageal dysphagia. Exclusions: structural lesions, mixed oropharyngeal dysphagia, recent proton pump inhibitor use, systemic sclerosis and Katz Activities of Daily Living < 6. HRM was performed and swallows classified per Chicago Classification v4.0. We report descriptive HRM metrics using logistic regression to identify independent predictors of normal HRM versus EMD.

**Results:**

Normal HRM was present in 57/176 (32.4%). Achalasia (44/176; 25%) and absent contractility (36/176; 20.5%) were the commonest EMDs; type II achalasia predominated. Median basal lower esophageal sphincter pressure and integrated relaxation pressure were lower in the normal-HRM group (15.3 ± 6.7 mmHg and 6.0 ± 4.0 mmHg) than in patients with EMDs (20.5 ± 23.5 mmHg and 11.0 ± 12.9 mmHg). On multivariable analysis, male sex and younger geriatric age band (66–70 years) were independently associated with EMD while Katz ADL and symptom duration were not. Correlations between age and continuous HRM metrics were weak.

**Conclusions:**

One-third of symptomatic older adults had normal contraction metrics on HRM; achalasia and absent contractility accounted for most pathologic findings. These data support early functional testing rather than attributing dysphagia to ageing alone. Prospective multimodal studies (panometry/impedance) are recommended to evaluate noncontractile mechanisms and refine referral algorithms.

## Introduction

Dysphagia is a common and consequential complaint in older adults, yet it is frequently attributed to “normal ageing” without objective functional evaluation. Age-related degeneration of the enteric nervous system can impair esophageal motor function even in the absence of a discrete motility disorder, which complicates clinical interpretation in geriatric patients (Wade and Cowen, 2004). When structural and oropharyngeal causes are excluded, persistent swallowing symptoms may reflect normal esophageal motility or an underlying esophageal motility disorder (EMD), both of which carry important downstream risks including aspiration pneumonia, malnutrition and increased healthcare utilization (Alagiakrishnan et al., 2013; Miller and Patterson, 2014). Dysphagia in older people also complicates medication administration and increases the risk of medication errors (Kelly et al., 2010; Kelly et al., 2011).

High-resolution manometry (HRM) is the current gold standard for assessing esophageal motor function and for classifying EMDs using the Chicago Classification (CC). Despite its growing clinical use, most classification schemes and normative datasets were developed in younger adults and do not explicitly address manometric patterns or diagnostic thresholds in geriatric populations. This gap is important because prior HRM and impedance-manometry studies in older cohorts report age-related changes in lower esophageal sphincter (LES) pressure, integrated relaxation pressure (IRP), distal contractile integral (DCI) and the frequency of simultaneous or weak contractions, but findings have been heterogeneous and sometimes contradictory (Grande et al., 1999; Besanko et al., 2011; Besanko et al., 2014; Shim et al., 2017).

Existing evidence therefore leaves two practical uncertainties for clinicians who care for older adults with dysphagia. First, what proportion of symptomatic geriatric patients will have normal contraction metrics on HRM versus a definable EMD? Second, do HRM metrics and the distribution of EMD subtypes differ across geriatric age bands or by sex in ways that should change diagnostic thresholds or referral practice? Answering these questions matters for primary-care clinicians and gastroenterologists deciding whether to pursue HRM, complementary physiologic testing (for example, FLIP or impedance), or empiric management.

To address these gaps, we conducted a cross-sectional HRM study in consecutive geriatric patients presenting with esophageal dysphagia. Our aims were to (i) classify EMDs using Chicago Classification v4.0, (ii) report esophageal pressure topographic metrics in this geriatric cohort, (iii) examine how the frequency of normal versus pathologic HRM findings varies across three geriatric subgroups (66–70, 71–75, >75 years), and (iv) evaluate correlations between age and key HRM metrics. The study was designed to provide clinically actionable data to inform referral decisions and to highlight physiologic areas (for example, distension-related mechanisms) that warrant prospective multimodal investigation.

## Material and methods

Our cross-sectional study was conducted jointly by the departments of physiology, geriatric medicine, internal medicine and medical gastroenterology at the gastrointestinal motility laboratory located in the department of physiology of a tertiary care center in a city in western India. The necessary Institutional Ethics Committee (IEC) approval was obtained (Number IEC/2022/159 dated 11 July 2022). All patients signed a written informed consent before entering the study. Data was collected from July 2022 until June 2025. The study was reported according to the STROBE statement criteria.

Men and women aged more than 65 years who complained of dysphagia and presented at the department of geriatric medicine and/or gastroenterology underwent a structured physician interview for clinical diagnosis of dysphagia. The clinical diagnosis was followed by upper gastrointestinal endoscopic and barium swallow evaluations. Those with structural lesions were excluded from the study. Otolaryngology opinion was obtained, and patients found with oropharyngeal causes of dysphagia were excluded. Patients with evidence of mixed oropharyngeal and esophageal dysphagia were also excluded. Patients with past history of abdominal surgery or procedures (like pneumatic dilatation and per-oral endoscopic myotomy), secondary causes of dysphagia, and a history of having consumed proton-pump inhibitors within the last 12 weeks were excluded. Since other common old age-related problems could lead to a false subjective perception of dysphagia and introduce bias in sample selection, the Katz Index of Independence in Activities of Daily Living (Katz ADL) was used and all patients with scores less than 6 were excluded from the study (Katz et al., 1970). Also excluded were patients who had consumed drugs affecting gut motility within seven days of HRM, systemic sclerosis (SSc) and other connective tissue disorders, and patients with myasthenia, Parkinsonism and other neurological disorders like cerebrovascular disease. Patients with gastro-esophageal reflux disease (GERD), laryngopharyngeal reflux disease (LPRD) and non-erosive reflux disease (NERD) were excluded from the study. We excluded patients with GERD, NERD, LPRD and Katz ADL < 6 to reduce confounding from reflux-related motility changes and severe functional dependence. While this improved the internal validity, it might limit generalizability to populations where such conditions are common. Patients with dysphagia but normal HRM topographic metrics were described as having normal HRM findings.

Sample size was calculated on the basis of the mean and standard deviation values of HRM metrics from a previous study on an Indian cohort using a 16-channel water perfusion system (Srinivas et al., 2018). Considering the given mean IRP of 13 mmHg with an SD of 10 mmHg, a mean difference of 5 mmHg, α = 0.05, and 80% power, the minimum required sample size was 150. Our final cohort of 176 exceeded this figure, ensuring adequate statistical power.

All the 176 patients underwent HRM in the morning of the appointed date after an overnight fast of at least eight hours. HRM was performed in the gastrointestinal motility laboratory in the department of physiology by the same gastrointestinal physiologist. A 16-channel water perfusion manometer (High-Resolution Manometry System, M/s Royal Melbourne Hospital, Parkville, Melbourne, Victoria, Australia) was used to obtain HRM data. Standardized 5 mL drinking water swallows were administered in the supine position (10 swallows) followed by upright position (five swallows), in accordance with the Chicago Classification v4.0 (CC v4.0) recommendations. The esophageal pressure topographic metrics were analyzed by the built-in software (Trace! Esophageal 1.3.4 (c), Royal Melbourne Hospital, Melbourne, Victoria, Australia) and swallows were classified as per the CC v4.0 guidelines.

The data was subjected to appropriate statistical analysis with the help of v23.0 of the International Business Machines Statistical Package for the Social Sciences. Numerical data were expressed as mean plus/minus standard deviation (mean ± SD), and categorical data were expressed as counts and percentages. Between-group comparisons used independent-samples t-tests or Mann–Whitney U tests for continuous data and χ² or Fisher’s exact tests for categorical data; paired comparisons used paired t-tests or Wilcoxon signed-rank tests. Correlations were assessed with Pearson’s or Spearman’s coefficients depending on distribution. The primary multivariable analysis was a logistic regression model to identify independent predictors of normal HRM versus EMD; candidate covariates (age band, sex, Katz ADL, symptom duration, key HRM metrics) were chosen *a priori* and multi-collinearity was checked. Effect sizes (Cohen’s *d*, odds ratios) with 95% CIs were reported for all primary comparisons, and two-sided tests were used with α = 0.05. No missing data were present, so multiple imputation was not required.

## Results

Normal HRM findings were reported in 57 patients (32.38%; males = 19, females = 38). **Table 1** summarizes the details of patients with normal and positive HRM findings and expresses the distribution of various EMDs. Achalasia was reported in 44 patients (25%; males = 29, females = 15), absent contractility in 36 (20.45%; males = 16, females = 20), fragmented peristalsis in 28 (15.91%; males = 20, females = 8), and ineffective esophageal motility (IEM) in 8 patients (4.55%; males = 4, females = 4). IEM was the rarest EMD affecting only 4.55% of elderly patients. There was no elderly patient with HRM evidence of distal esophageal spasm and jackhammer esophagus. Type II achalasia was the commonest subtype (31 patients, 70.45% of all achalasia cases; males = 22, females = 9), followed by type I (9 patients, males = 7) and type III (4 patients, male = 1). The details of achalasia subtypes between the genders are depicted in **Figure 1**. Various HRM metrics measured in patients with normal HRM findings are summarized in **Table 2**, whereas **Figure 2** compares the normal versus positive HRM findings in elderly males and females belonging to different geriatric age sub-groups, namely from 66 to 70 years, 71 to 75 years and above 75 years. Differences in the ratio of normal to positive HRM findings were observed in the three different age sub-groups. 12 out of 26 (46.15%) patients belonging to the >75 years age group had normal HRM findings despite dysphagia, which was higher than the normal HRM findings in the 66–70 years group (38.33%). Pearson’s correlation coefficients between age and various HRM metrics are shown in **Table 3**, which revealed weak correlations between ageing and the measured HRM metrics.

**Table 1:** Normal and positive HRM findings as per gender in the geriatric age sub-groups, based on CC v4.0.

HRM swallow classification (CCv4.0)	Geriatric age sub-groups (years)			Total	Grand total
	66-70	71-75	>75		
Achalasia (males)	12	10	7	29	44
Achalasia (females)	7	7	1	15
Absent contractility (males)	7	5	4	16	36
Absent contractility (females)	13	0	7	20
Fragmented peristalsis (males)	10	5	5	20	28
Fragmented peristalsis (females)	3	3	2	8
Ineffective esophageal motility (males)	3	1	0	4	8
Ineffective esophageal motility (females)	4	0	0	4
EGJOO (males)	0	1	0	1	3
EGJOO (females)	2	0	0	2
**Total EMDs**	**61**	**32**	**26**		**119**
**Total EMDs (males)**				**70**
**Total EMDs (females)**				**49**
Normal motility (males)	8	7	4	19	X
Normal motility (females)	15	15	8	38
**Total normal motility**	**23**	**22**	**12**	**57**

Figures in bold indicate total number of patients belonging to a particular group.

**Figure 1:**
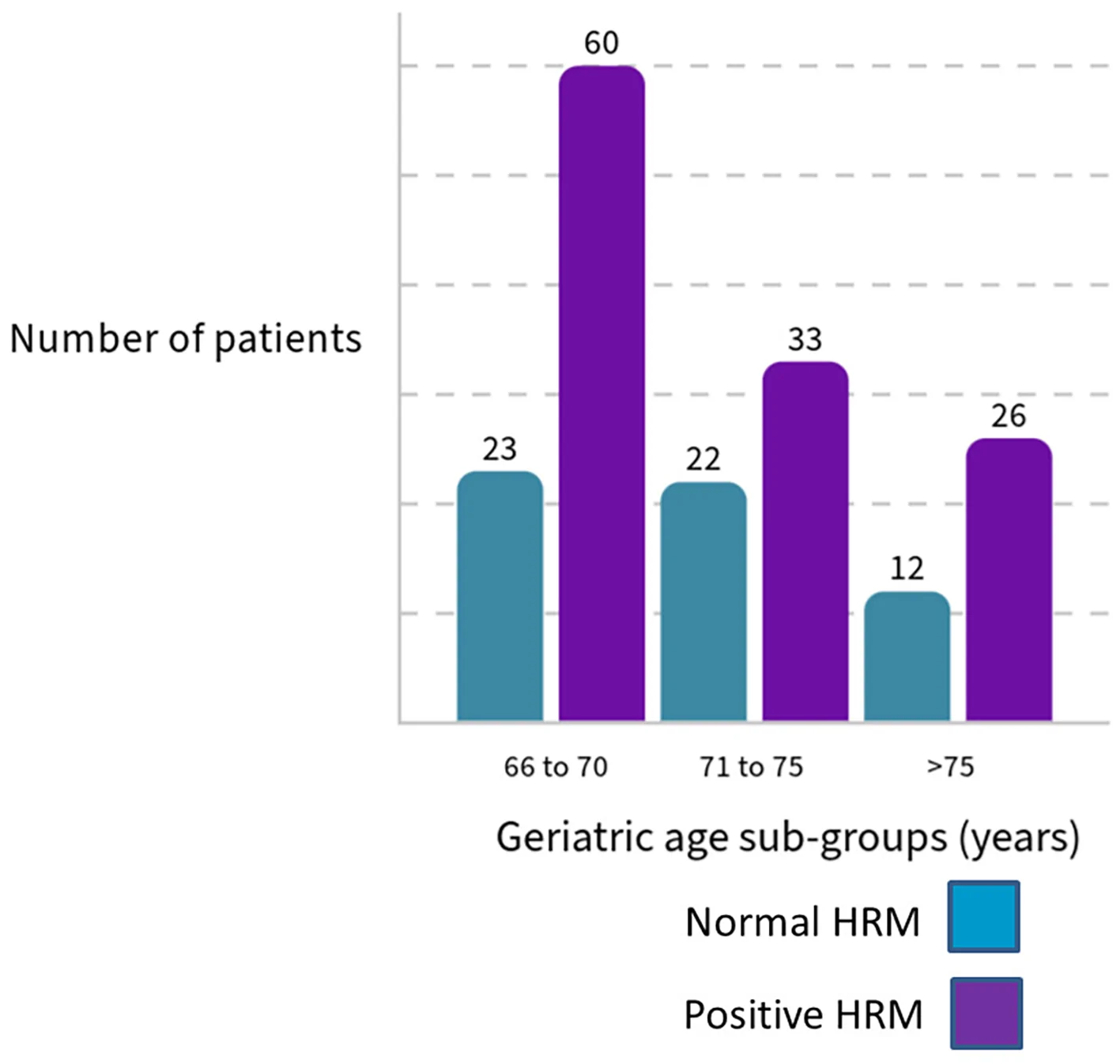
Gender wise distribution of achalasia sub-types among the elderly.

**Table 2:** Comparison between HRM metrics of patients having normal HRM findings and those having EMDs.

HRM metrics	Normal HRM	Positive HRM
Basal maximum (mmHg)	15.275 (± 6.71)	20.527 (± 23.48)
Basal minimum (mmHg)	9.916 (± 5.47)	13.823 (± 17.75)
Basal median (mmHg)	12.939 (± 6.12)	16.981 (± 19.59)
LES nadir (mmHg)	5.011 (± 3.84)	10.476 (± 13.83)
IRP (mmHg)	6.002 (± 3.97)	11.034 (± 12.94)
DCI supine (mmHg.s.cm)	1155.6140 (± 720.99)	941.3827 (± 1411.02)
DCI upright (mmHg.s.cm)	1242.3667 (± 824.97)	1225.2357 (± 1824.45)

Data have been expressed as mean ± standard deviation.

**Figure 2:**
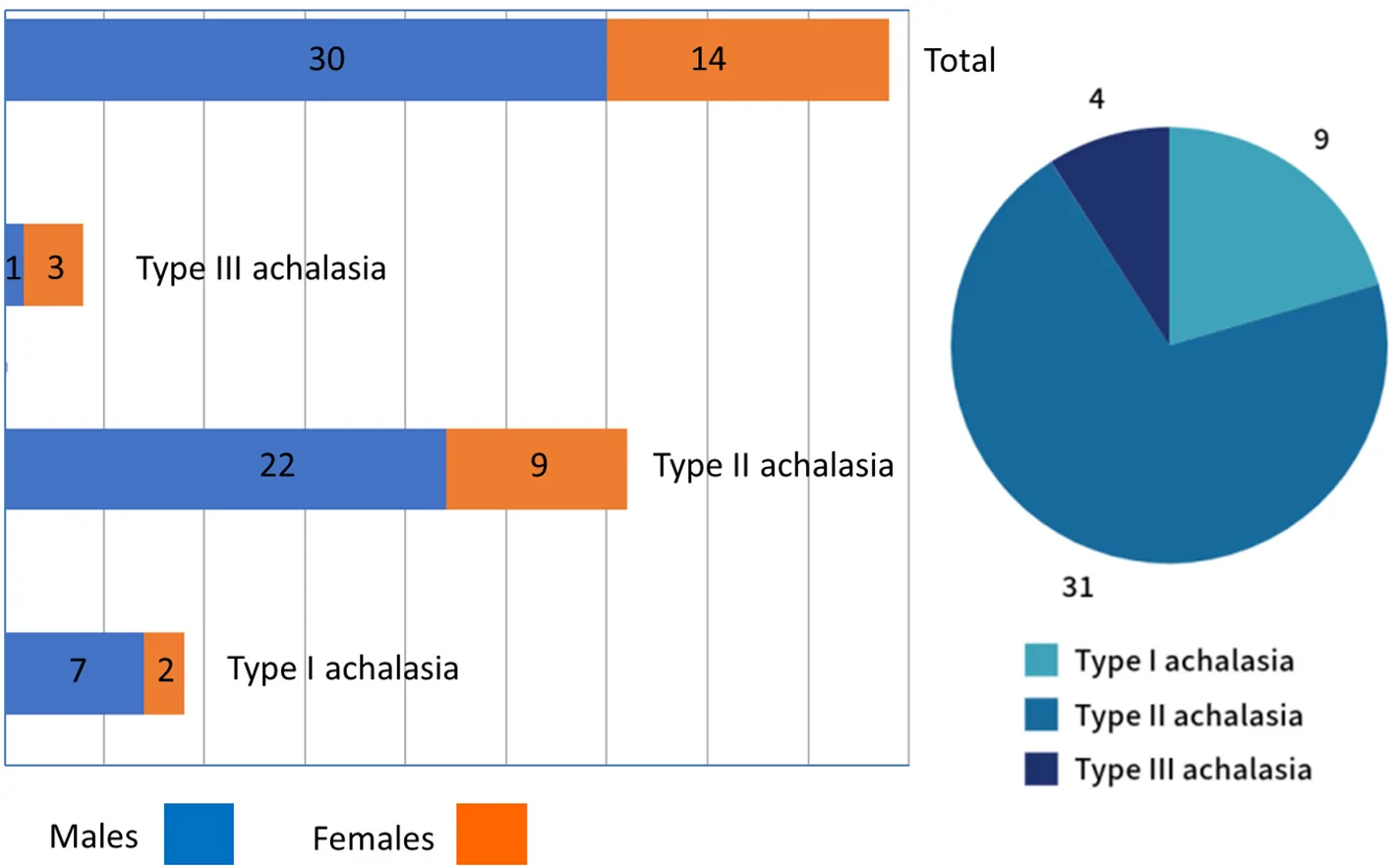
Comparative depiction of normal versus positive HRM findings in elderly males and females belonging to different geriatric age sub-groups.

**Table 3:** Pearson’s correlation coefficients between age and various HRM metrics. No strong correlations were observed in this study.

	Basal maximum	Basal minimum	Basal Median	LES nadir	IRP	DCI supine	DCI upright	DCI area supine	DCI area upright
Age	0.156^*^	0.202^**^	0.171^*^	0.227^**^	0.130	0.165^*^	0.117	0.128	0.092

*Correlation is significant at 0.05 (two-tailed).

**Correlation is significant at 0.01 (two-tailed).

Values between 0.7 and 1: Strong correlation.

Values between 0.3 and 0.7: Moderate correlation.

Values between 0 and 0.3: Weak correlation.

On multivariable logistic regression, male sex (OR 1.92, 95% CI 1.08–3.41, p = 0.024) and younger geriatric age band (66–70 years; OR 2.07, 95% CI 1.15–3.72, p = 0.015) emerged as independent predictors of esophageal motility disorders. By contrast, Katz ADL (OR 1.01, 95% CI 0.82–1.25, p = 0.89) and symptom duration (OR 1.09, 95% CI 0.91–1.31, p = 0.34) were not significantly associated with abnormal HRM findings. Because all 176 cases had complete data for the included covariates, analyses were performed on the full dataset without imputation. The complete regression model is presented in **Table 4**.

**Table 4:** Multivariable logistic regression model including all candidate covariates (age band, sex, Katz ADL, symptom duration).

Variable	Odds Ratio (OR)	95% CI	p-value
Male sex	1.92	1.08–3.41	0.024
Age 66–70 vs >75	2.07	1.15–3.72	0.015
Katz ADL	1.01	0.82–1.25	0.89
Symptom duration	1.09	0.91–1.31	0.34

Odds ratios (OR), 95% confidence intervals (CI), and p-values are shown. NS = not significant. All 176 cases had complete data; no imputation was required.

## Discussion

This is the first study which has measured and described various esophageal pressure topographic metrics among elderly Indians with esophageal dysphagia. We found that almost one-third of elderly patients with dysphagia had normal HRM findings. Available HRM systems currently measure only the contraction characteristics of the esophagus, which is only a part of the entire process of deglutition. These systems cannot measure the other part, which is relaxation or distension, because the esophageal body does not have a resting tone which the LES has (Mittal and Zifan, 2024). Hence, quite a few patients would be found with normal HRM findings despite having dysphagia. Distension related issues might explain the dysphagia in such patients (Zifan et al., 2024). Whether the percentage of elderly patients with normal HRM findings despite dysphagia is similar to that among younger people is a matter that lacks supportive evidence. Our study also stands out because we further divided our patients into three geriatric age sub-groups and found that the ratio of normal to positive HRM findings in them were different. Thirty-eight percent (38.33%) of patients aged 66–70 had normal HRM studies; this proportion increased to 66.67% in the 71–75 group and declined to 46.15% in those >75 years. The pattern is therefore non-linear rather than steadily increasing with age. The unexpectedly high proportion of normal HRM in the 71–75 subgroup suggests heterogeneity in symptom perception, referral patterns, or underlying pathophysiology across geriatric bands; by contrast, the >75 group again shows a substantial—but lower—rate of functional dysphagia. These observations argue for cautious interpretation of age effects and for considering complementary tests when HRM is normal. An interesting observation was that the number of elderly women having functional esophageal dysphagia was twice as much as elderly men. It has been suggested that men and women have different normal HRM metrics (Jung et al., 2015) and gender-specific values may be required for appropriate diagnosis (Vega et al., 2010), which was refuted later by a 2017 Brazilian study (Costa and Dantas, 2017). However, these studies were performed on healthy individuals and not on those with functional dysphagia, and neither did they include geriatric participants. An Australian audit of 165 manometry reports of subjects aged more than 65 years revealed that women of older age groups were referred more commonly for HRM, than were the older men (Andrews et al., 2009). Our study, however, had almost equal gender-wise referrals, with 89 men and 87 women, respectively, and yet normal HRM findings were twice as common among elderly women having dysphagia. Esophageal hypervigilance, wherein individuals pay excessive attention to the sensations in the esophagus has been described in literature (Carlson et al., 2020). However, our data could not establish a causality, and this interpretation should be considered explanatory. Taken together, the key results of our study are that nearly one-third of elderly patients with dysphagia had normal HRM findings, while achalasia and absent contractility were the predominant motility disorders, with type II achalasia being most frequent. Male sex and younger geriatric age sub-groups were independent predictors of EMD, whereas correlations between age and HRM metrics were weak.

Median basal LES pressure in the present study was 15.275 ± 6.71 mmHg among patients with functional esophageal dysphagia, which was lesser than an Egyptian study conducted on healthy subjects older than 65 years (Abdelghani et al., 2023). However, data on elderly patients with functional esophageal dysphagia are sparse. As stated earlier, LES pressures in healthy people above the age of 65 years have been found to be lower than those in younger adults (Shim et al., 2017) and LES relaxation has been found to be impaired in the elderly with dysphagia (Besanko et al., 2011). Our study did not find any significant alterations to these values. Mean IRP value in the present study was 6 ± 3.97 mmHg, which was lower than (13 mmHg) that reported among younger healthy Indians, as well as the values used in the Chicago Classification (Srinivas et al., 2018). Thus, considering normal values of IRP found in the present study, geriatric patients with dysphagia having normal HRM findings had adequate esophago-gastric junction relaxation during normal swallows. Multivariate analysis has shown statistically significant positive correlation (co-efficient of 0.12; p=0.001) between DCI and age (Djinbachian et al., 2021), even though one study did find a lower DCI in the elderly (Shim et al., 2017). In our study, the mean DCI among the elderly with normal HRM were 1155 ± 721 mmHg.s.cm in the supine position and 1242 ± 367 mmHg.s.cm in the upright position. Among those with positive HRM findings, mean DCI was 941 ± 1411 mmHg.s.cm in the supine position and 1225 ± 1824 mmHg.s.cm in the upright position. Younger Indian healthy adults (mean age = 30 years) have been found to have lower IRP (13 mmHg) as well as DCI values (350–4500 mmHg.s.cm) when compared to those used in the Chicago Classification (Srinivas et al., 2018), but higher than that found in the elderly patients of the present study. Our study could not establish a strong relationship between ageing and HRM metrics among the elderly Indian population upon applying the Pearson’s correlation coefficient. Therefore, it would be reasonable to infer that ageing per se may not lead to statistically significant changes in the HRM metrics. Our study did not find a single patient with raised contraction vigor, and hence hypercontractile motility disorders and distal esophageal spasm. However, such EMDs do occur in the elderly and may even be commoner (10.4% versus 9.2%) than younger adults (Kunen et al., 2020). The same report also supports the present study wherein hypocontractile EMDs were commoner in the Indian elderly when compared with hypercontractile ones. In our study absent contractility (30.25%) and fragmented peristalsis (25.7%) were common, although not as much as ineffective esophageal motility (IEM). IEM was the least frequent EMD in our cohort (4.55%). Absent contractility is also the commonest HRM finding in SSc and has been found to affect SSc patients as old as 68 years of age, but without accompanying dysphagia (Kartik et al., 2020). IEM is common among patients with gastro-esophageal reflux disease (GERD), including elderly patients as old as 70 years (Bhattacharya et al., 2024), although the disease may not always present with dysphagia. IEM is also the most common HRM finding in laryngopharyngeal reflux disease (LPRD) and may be present with or without accompanying dysphagia. However, the present study did not include LPRD patients since HRM is not its frontline diagnostic tool (Datta et al., 2011). Achalasia was the most common EMD found in the elderly. Achalasia is commonly diagnosed between 20 to 40 years of age (Eckardt et al., 2008). However, it has also been found to be far more common among the elderly residing in north-eastern Italy, in the region around Verona and Venice (Gennaro et al., 2011), suggesting that there might be a second peak in old age. In our study, more than 80% of geriatric achalasia cases were aged between 66 and 75 years, with less than one-fifth aged more than 75 years. In a study conducted in the same demographic area as ours, but in adults aged 18 to 60 years, 31.7% patients were type I achalasia, 43.9% were type II and 24.4% were type III (Kumar et al., 2024). However, in the same demographic area, these values for the geriatric age group were 20.45% for type I achalasia, 70.45% of type II and 9.1% of type III. Clearly, type II achalasia was almost three times more in the elderly and type III was almost two and half times less, as compared to the younger population belonging to the same demographic area in western India, which is an important finding of the present study. Although the incidence of achalasia is similar in males and females (Eckardt et al., 2008), we found that achalasia was twice as common in Indian geriatric males as in females. However, EGJOO was a rare phenomenon in the present study, with only three cases (< 2%) diagnosed on HRM. All EGJOO cases fulfilled the CCv4.0 diagnostic criteria, including the accompanying clinically relevant symptom of dysphagia and timed barium esophagogram. The average age for EGJOO has been mentioned to be between 51 and 69 years (Lynch et al., 2017), and two out of three cases were aged below 70 years.

Our study has limitations. Despite using the Katz ADL to reduce bias, residual confounders affecting symptom perception in older adults could not be fully excluded, and recruitment was symptom-based rather than population-based. We also report single-center data from western India, which may limit generalizability to other regions and to frailer elderly populations. We did not perform routine distension or impedance testing, so non-contractile mechanisms of dysphagia may have been missed. We also acknowledge that the geriatric age sub-groups were more of a pragmatic decision rather than one based on evidence; and subgroup comparisons should be interpreted with due caution. Although solid-state catheters are now the international standard, 16-channel water-perfused systems remain widely used in India. They also fulfil CCv4.0 requirements for diagnostic classification, though their lower spatial resolution compared with solid-state systems is a limitation that we acknowledge (Sharma and Yadlapati, 2022). We used the Katz ADL to exclude patients with severe dependence, thereby reducing bias from functional impairment. However, Katz ADL does not assess frailty, sarcopenia, muscle strength, or nutritional status, which may be more closely associated with swallowing dysfunction than chronological age. The absence of these measures is an important limitation of our study.

Nevertheless, this relatively large geriatric HRM series provides novel regional data: a substantial proportion of symptomatic older adults had normal HRM (functional dysphagia), while achalasia and absent contractility accounted for most pathologic findings. These results emphasize that dysphagia in older patients should not be reflexively attributed to ageing; early, structured evaluation and appropriate referral can identify treatable disorders. Future multicenter, multimodal studies are needed to validate these findings and refine primary-care referral pathways.

## Data Availability

The original contributions presented in the study are included in the article/supplementary material. Further inquiries can be directed to the corresponding author.
